# Functional Class in Children with Idiopathic Dilated Cardiomyopathy. A pilot Study

**DOI:** 10.5935/abc.20160066

**Published:** 2016-06

**Authors:** Aline Cristina Tavares, Edimar Alcides Bocchi, Guilherme Veiga Guimarães

**Affiliations:** Instituto do Coração (InCor), Hospital Sírio-libanês (HSL), São Paulo, SP - Brazil

**Keywords:** Heart Failure, Cardiomyopathy, Dilated / mortality, Stroke Volume, Child, Pilot Projects

## Abstract

**Background:**

Idiopathic dilated cardiomyopathy (IDCM), most common cardiac cause of pediatric deaths, mortality descriptor: a low left ventricular ejection fraction (LVEF) and low functional capacity (FC). FC is never self reported by children.

**Objective:**

The aims of this study were (i) To evaluate whether functional classifications according to the children, parents and medical staff were associated. (iv) To evaluate whether there was correlation between VO_2_ max and Weber's classification.

**Method:**

Prepubertal children with IDCM and HF (by previous IDCM and preserved LVEF) were selected, evaluated and compared. All children were assessed by testing, CPET and functional class classification.

**Results:**

Chi-square test showed association between a CFm and CFp (1, n = 31) = 20.6; p = 0.002. There was no significant association between CFp and CFc (1, n = 31) = 6.7; p = 0.4. CFm and CFc were not associated as well (1, n = 31) = 1.7; p = 0.8. Weber's classification was associated to CFm (1, n = 19) = 11.8; p = 0.003, to CFp (1, n = 19) = 20.4; p = 0.0001and CFc (1, n = 19) = 6.4; p = 0.04).

**Conclusion:**

Drawing were helpful for children's self NYHA classification, which were associated to Weber's stratification.

## Introduction

Idiopathic dilated cardiomyopathy (IDCM) - characterized by left ventricular dilatation and systolic dysfunction of undetermined cause,^1-3^ has a high incidence among the pediatric population^4^ and an unfavorable outcome,^2,5,6^ and is thus a target for reasearch.^1^

To date, it is known that the only predictors of death or cardiac transplantation in children with IDCM are a low LVEF and low functional capacity.^7^

LVEF is easily measured by echocardiography.^8^ Functional capacity, in turn, may be determined using peak oxygen consumption (VO_2_) in the cardiopulmonary exercise test (CPET)^9,10^ or scales representing the functional class.^3,11,12^ CPET findings provide an objective assessment of the functional capacity,^9,13^ whereas the scales represent a subjective assessment.^13^

However, the scales are not always related to the objective values of CPET,^13,14^ and this may impair the communication between parents and the medical team, the stratification, and treatment. Thus, the objective of this study is to fill this gap and evaluate whether there is a correlation between the objective functional capacity (by peak O_2_ consumption - peak VO_2_ ) and the functional class as proposed by the family, the medical team and the child itself, and whether there is a correlation between peak VO_2_ and Weber stratification.^12^

## Methods

### Sample

This is a pilot, cross-sectional, prospective, randomized, consecutive study. Children of both genders with IDCM and children with HF with preserved LVEF (secondary to previous IDCM) were selected from the outpatient clinic of the Medical Unit of Pediatric Cardiology and Congenital Heart Defects of *Instituto do Coração* - InCor, *Hospital das Clinicas da Faculdade de Medicina da Universidade de São Paulo* - HCFMUSP.

The inclusion criteria were: (i) patients diagnosed with current IDCM or HF for previous IDCM with preserved LVEF; (ii) patients clinically stable; (iii) patients receiving drug therapy continuously for the past 3 months; (iv) older than 5 years;^15,16^ (v) age equivalent to the prepubertal phase -*Tanner-*Whithouse scale stages 1 to 3;^17^ (vi) previous echocardiographic study performed at least 6 months earlier.

Children with complex ventricular arrhythmias or atrial fibrillation; in the postoperative recovery period; with neuromuscular, renal or pulmonary diseases; with diabetes mellitus; and/or those who refused to participate in the study or in the assessments were not included.

The children, as well as their guardians (as established in articles 1634,^18^ and 1852,^19^ subsection V of the Civil Code, and in Law 8069/90 and 10406/2002),^20^ included in any of the groups, were given information on the objectives of the research and the tests participants should undergo. In addition, all children participating and their parents or guardians were informed that the children should keep taking their regular medication throughout the study. All children or guardians gave written informed consent to participate.

The children were included according to the inclusion criteria and were assessed provided they were cleared by the medical team.

### Assessments

All children were assessed as regards their functional class, anthropometric data and CPET.

### Functional class

The modified functional classification used was adapted from a functional classification previously described elsewhere and applied in studies assessing children with cardiomyopathies,^3,21^ as follows:

Class I - Heart disease with no limitation of physical activities. Schoolchildren are able to attend physical education classes until the end.

Class II - Slight limitation of physical activities. Comfortable at rest, but ordinary activities may result in tachycardia, fatigue or dyspnea. Schoolchildren attend physical education classes, but are unable to stay until the end.

Class III - Marked limitation of physical activities. Less than ordinary activities, such as walking less than a block, may cause fatigue, tachycardia or dyspnea. Schoolchildren are unable to attend physical education classes.

Class IV - Unable to carry on any physical activity without discomfort. Symptoms are present at rest and increase during activity.

**Figure 1:**
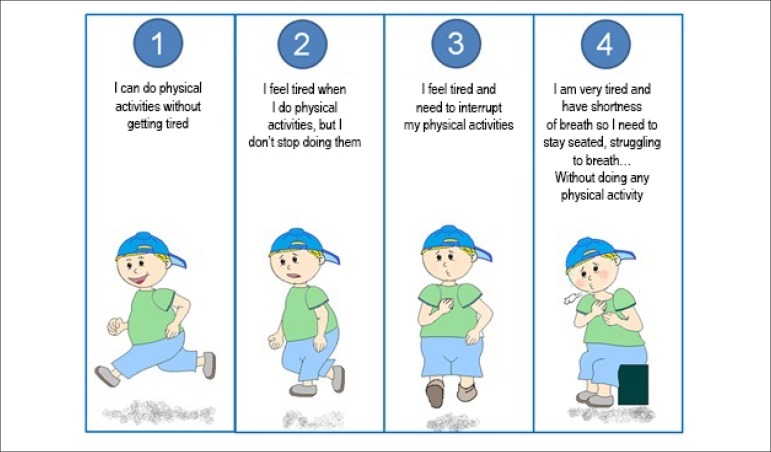
Functional class for male children.

**Figure 2:**
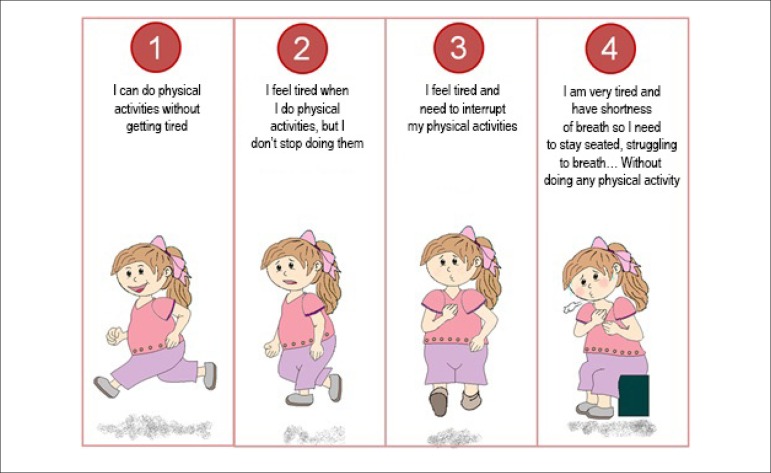
Functional class for female children.

Based on this description, a graphic representation of the four functional classes was elaborated by this study's author, both for male and female children (Figures 1 and 2, respectively), so that the guardians and the children could use it. In order to make these drawings, the image taken into consideration was the one with which children in the same age range as those participating in the study could identify themselves.

Initially, the physician following up the children would give his/her opinion on which functional class the children were in. This baseline assessment was made without the presence of the guardians or the children themselves. This information was expressed as functional class according to the medical team (FCm).

Next, the parents or guardians would give their opinion on the functional class the children were in, according to figures 1 and 2. This classification was made without the presence of the physicians or even the children themselves. This information was expressed as functional class according to parents or guardians (FCp).

Later, the children would perform a self-assessment of their functional class using the graphic representation (Figures 1 and 2). This self-perceived functional class was expressed as functional class according to the children themselves (FCc).

### Anthropometric data

Data on age, gender, height, body mass and body mass index (BMI) were collected.

### Echocardiographic data

Analysis of the cardiac function using echocardiography was considered for studies performed up to six months prior to inclusion.

Echocardiographic studies were performed according to recommendations from the guidelines for the pediatric population, using the Teicholz method.^22^ Data on LVEF, end-diastolic left ventricular size, end-systolic left ventricular diameter, and left ventricular wall thickness were collected. Size and thickness values were corrected for body surface area (BSA) using a formula appropriate for children weighing more than 10 kg, as follows: BSA = (weight *4 +7) / (weight + 90),^23^ in which weight is expressed in kg.

Children, whose medical record contained a previous echocardiographic study performed no later than six months prior to the collection of the other data, would have their echocardiographic data retrieved from that previous study. Children with no previous echocardiographic study underwent the test, from which the data were further collected.

### Cardiopulmonary exercise test

The children underwent a cardiopulmonary exercise test (CPET) in a programmable treadmill (Marquette series 2000, Marquette Electronics, Milwaukee, WI, USA), according to the modified Balke ramp protocol.^21,24-26^

CPET was performed two hours after a caffeine-free light meal, in a room with controlled temperature (21°C to 23°C), after a 2-minute rest, in the upright position on the treadmill.^25^

During the beginning of the resting, exercise, and recovery periods, the children had their pulmonary ventilation as well as oxygen and carbon dioxide concentrations in the inhaled and exhaled air volumes continuously monitored (Sensormedics, model Vmax 229, Yorba Linda, CA, USA), breath by breath. During CPET, continuous 12-lead heart rhythm monitoring was performed (Marquette MAX 1, Marquette Electronics, Milwaukee, WI, USA) and systemic blood pressure was measured every minute (HP68S Hewlett-Packard multiparameter monitor, USA, or HP M1008B Hewlett-Packard oscillometric blood pressure transducer, USA).^24-26^

Criteria for exercise termination were the absolute indications recommended by the ACC/AHA Guidelines Update For Exercise Testing, when exhaustion was reached (respiratory quotient > 1.0)^25^ or in the presence of signs or symptoms that could result in cardiac injury, such as angina, headache, dizziness, syncope, excessive dyspnea, fatigue, ST-segment depression or elevation greater than 3 mm, arrhythmia, supraventricular or ventricular tachycardia, atrioventricular block or progressive decrease in blood pressure (BP).^25^

### Statistical Analysis

The statistical analysis was carried out using the SPSS 12.0 software program for Windows (SPSS Inc., Chicago, IL, USA).

The Shapiro-Wilk test was used to check the normality of data in the population.

Patient demographics were expressed in a descriptive manner, in absolute numbers, percentages or mean and standard deviation. Functional classes were presented as absolute numbers. Quantitative variables regarding the cardiopulmonary exercise test were expressed as mean and standard deviation.

The chi-square test (χ^2^) was used to analyze the association between categorical variables of the functional class, as assessed by the medical team, guardians and children.

The Pearson correlation coefficient was used for normal data, and the Spearman correlation, for non-parametric data, in order to correlate quantitative data. These correlations were interpreted as directly proportional (if +) or inversely proportional (if -), and weak (if 0.1 to 0.29), moderate (if 0.3 to 0.59), strong (if 0.6 to 0.79), very strong (if 0.8 to 0,99) or perfect (if 1).^27^

## Results

Initially, 77 children were screened to comprise the sample. Only 31 met all inclusion criteria; however, only 19 agreed to participate in the study. The post-hoc Bonferroni test showed that there was no significant effect for gender among the children.

None of the 19 children presented any hemodynamic instability during the cardiopulmonary exercise test.

The children were using the following medications: acetyl salicylic acid, captopril, carvedilol, digoxin, enalapril, spironolactone, furosemide, and topimarate.

Table 1 shows the characterization of the overall sample, with details on its demographics and echocardiographic data.

**Table 1:** Sample characterization

		Total (19)
**Demographics**		
Age (years)		8.7 ± 1.9
Gender (F/M)		10/9
Body mass (kg)		30.7 ± 8.5
Height (m)		1.26 ± 0.45
BMI (kg/m^2^)		30.7 ± 8.5
BSA (m^2^)		111.2 ± 41.5
**Echocardiographic data**		
	– LVEF (%)	46.7 ± 13.8
	– Systolic LV size	48.3 ± 9.8
	– Diastolic LV size	37.5 ± 12.2
	– Relative LV wall thickness	0.26 ± 0.06

BSA: body surface area; IDCM: children with idiopathic dilated cardiomyopathy and LVEF < 40%; LVEF: left ventricular ejection fraction; HF: heart failure; BMI: body mass index.

According to the medical team, 13 children were classified as FC I, five as FC II, one as FC III, and none as FC IV.

According to parents, 13 children were classified as FC I, four as FC II, one as FC III and one as FC IV.

According to the self-assessment, 11 children classified themselves as FC I, six as FC II, two as FC III. No children classified themselves as FC IV.

Table 2 shows FCm, FCp, FCc, and peak VO_2_ reached in the cardiopulmonary exercise test for each participant.

**Table 2:** Cardiopulmonary exercise test data

		All (19)
**SBP (mmHg)**		
	– rest	102.2 ± 12.4
	– peak	120.5 ± 18.1
**DBP (mmHg)**		
	– rest	59.2 ± 10.6
	– peak	69.7 ± 13.7
**HR (bpm)**		
	– rest	91.2 ± 10.8
	– maximum	162.1 ± 18.7
VO_2-peak_ (mL/kg/min)		25.5 ± 6.7
VE/VCO_2_ slope		37.4 ± 6.4
RER		1.02 ± 0.04
PetO2		54.3 ± 30.3
Time (min)		10.9 ± 4.3

HC: heart rate; DBP: diastolic blood pressure; SBP: systolic blood pressure; PetO2: pressure of end-tidal O_2_; RER: respiratory exchange ratio; VE/VCO_2_ slope: slope of the line between ventilation (VE) and carbon dioxide production (VCO_2_), VO_2_ peak: peak oxygen consumption.

The chi-square test showed an association between FCm and FCp (1, n = 31) = 20.6; p = 0.002. No significant association was found between FCp and FCc (1, n = 31) = 6.7; p = 0.4. FCm and FCc were not associated either (1, n = 31) = 1.7; p = 0.8.

**Figure 3:**
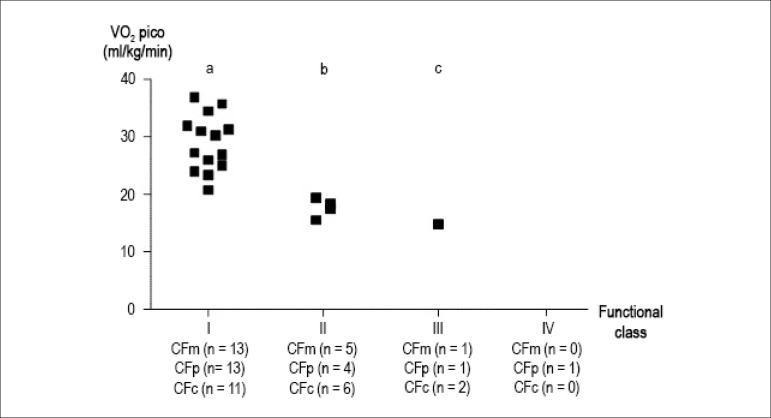
Functional class, Weber classification, and peak oxygen consumption. c: child; FC: functional class; m: medical team; p: parents or guardians; VO_2_ peak: peak oxygen consumption. ^a^p = 0.003; ^b^p = 0.0001; ^c^p = 0.04.

According to the peak VO_2_ found in CPET, Weber classification was significantly associated with the three functional classes described in this study, using the χ^2^ test: Weber classification and FCm (1, n = 19) = 11.8; p = 0.003; Weber classification and FCp (1, n = 19) = 20.4; p = 0.0001; Weber classification and FCc (1, n = 19) = 6.4; p = 0.04. (Figure 3).

Children from the sample reached 84% of the maximum HR, according to the formula proposed by Tanaka (maximum HR = 208 - [0.7 x age]),^28^ with this maximum HR being approximately 35 bpm lower than that proposed.

Peak VO_2_ and LVEF values showed a weak non-significant correlation between each other (r = 0.27; p = 0.25). Likewise, LVEF was not related to the other data obtained from CPET.

Finally, Table 3 shows all data from the present study, including data on the functional classes (FCm, FCp, FCc) and peak VO_2_ as measured by cardiopulmonary exercise test, for each study subject.

**Table 3:** Data on functional classes and peak oxygen consumption on cardiopulmonary exercise test

Subject	FCm	FCp	FCc	VO_2_ peak
1	1	1	1	34.6
2	2	1	3	32
3	2	1	1	17.6
4	1	1	1	30.3
5	2	1	2	27
6	2	2	1	25
7	1	1	2	23.4
8	1	4	2	15.6
9	1	1	3	31
10	2	2	1	36.9
11	1	1	1	35.8
12	1	2	1	26
13	1	2	1	15.6
14	1	1	2	14.8
15	1	1	2	27.3
16	1	1	1	24
17	1	1	2	18.4
18	1	1	1	30
19	1	1	1	31.3

FCc: functional class according to the children themselves. FCm: functional class according to the medical team; FCp: functional class according to parents or guardians; VO_2_ peak: peak oxygen consumption.

## Discussion

Although the study sample had a small number of participants, our findings show that the cardiopulmonary exercise test is safe in the populations described; that peak VO_2_ findings are related to the stratification data using Weber classification;^12^ and that drawings can be an additional resource for the assessment of children with IDCM and HF (for previous IDCM) with preserved LVEF.

As regards the anthropometric data, all children enrolled were in the prepubertal phase,^17^ thus there was no influence of hormones on the results obtained.^29^

Although all children included in the present study had been in the same age range in which linear growth occurs (from 7 to 11 years of age),^30^ children with IDCM were shorter than those with HF. This may have resulted from low weight gain during childhood^31^ because of a low systemic supply secondary to impaired cardiac output that children with more severely affected hearts show.^32^

The medications used were consistent with those described in the literature for the treatment of IDCM or HF in the pediatric population, including angiotensin-converting enzyme inhibitors (ACEI),^33^ betablockers and diuretics^1^.^26^

The cause of short stature in children with IDCM and of high drug doses may be similar to that of nocturnal dip. The latter, in turn , is related to severity of symptoms and greater sympathetic activity.^34^ In this regard, further studies are probably necessary to establish these associations.

Like for adults, exercise tolerance is known to be predictive of mortality in children with heart failure.^7^ Additionally, the experience with cardiopulmonary exercise test^21,25^ in healthy children^16^ and in those with HF for IDCM^25^ older than 6 years,^16,25,32^ show that the cardiovascular and metabolic responses are similar to those observed in adults with the same clinical characteristics.^17^

In our CPET assessments, we observed that both groups of children with IDCM and those with HF with preserved LVEF are unable to reach the maximum age-predicted HR in the exercise test. These findings are corroborated by results of studies conducted in adults with HF^35^ and in children with IDCM,^17^ in which 80% of the maximum HR in the mean for age was reached, and are similar to those found in the present study, in which the values are between 82% and 84% of the maximum HR.

Peak VO_2_ values found in the present study were different in the two groups. This probably resulted from the fact that peak VO_2_ is believed to occur between 13 and 14 years of age,^16,21,29^ i.e., the parameters related to this indicator are expected to be rising during the prepubertal period, phase in which the participants were assessed.^29,36^ Although a systematic review by the present study's author had shown that peak VO_2_ values in prepubertal girls are, on average, 20% lower than those found in prepubertal boys,^37^ because of the influence of hormones and body fat,^37,38^ this finding was not observed here after post-hoc Bonferroni test. We can suppose that the small sample size had a negative influence on the analysis of this variable.

Even with peak VO_2_ values lower than expected,^37^ all children reached the maximum test according to the criteria of exercise termination mentioned by ACC/AHA Guidelines Update for Exercise Testing,^39^ because the modified Balke protocol used is appropriate to the study population, and the protocol-demanded response to exercise is similar to the physiological response to exercise in children. That is, the time to reach 50% of peak VO_2_ values in children is shorter than that for adults; children are less dependent on the glycogenic pathway to meet the demands than adults; the use of fatty acids as an energy source is greater during childhood; and children show lower levels of blood lactate (which makes it more difficult to reach exhaustion), lower pulmonary ventilation (VE, L/min) and lower carbon dioxide production (VCO_2_, mL/min).^29,39^

Since the information on the functional class as assessed by the children, their guardians and the medical team was not correlated, the data prove to be subjective, which is corroborated by previous studies.^13,14^ However, it was correlated with peak VO_2_ values on CPET,^40^ according to Weber's criteria, which are very frequently used for stratification and prognosis in adults.^12^ Since, to date, no such prognostic assessment exists in the scientific literature regarding children with IDCM and HF, the measurement will probably continue to be subjective, corroborating previous findings from 2001, in which objective values on CPET did not correlate with the functional class as assessed by the medical team.^13^

## Conclusion

Peak VO_2_ peak are related to stratification data by Weber classification, and the drawings shown to prepubertal children may be an additional resource for the assessment of children with IDCM and HF (for previous IDCM) and preserved LVEF.
